# A discursive analysis concerning information on “ADHD” presented to parents by the National Institute of Mental Health (USA)

**DOI:** 10.3402/qhw.v11.30938

**Published:** 2016-04-05

**Authors:** Soly Erlandsson, Linda Lundin, Elisabeth Punzi

**Affiliations:** 1 Department of Social and Behavioral Studies, University West, Trollhättan, Sweden; 2 Department of Psychology, Gothenburg University, Gothenburg, Sweden

**Keywords:** Attention-deficit hyperactivity disorder, discourse, medicalization, children's behavior, suffering

## Abstract

A discourse analysis was performed based on an online document under the headline: “What is Attention Deficit Hyperactivity Disorder (ADHD, ADD)?” published by the National Institute of Mental Health (NIMH), USA. Three parts of the document were analysed: (1) The introductory part, as this sets the tone of the whole text. (2) Parts of the text that were specifically addressed to parents. (3) Etiology and pathology of “ADHD” with reference to a number of different symptoms and behaviors. Inattention and hyperactivity are presented in the document as a floating spectrum of symptoms caused by “ADHD.” Other factors of importance for children's development, that is, early attachment, close relationships, previous experiences, culture, and contexts are ignored. Children who are perceived as inattentive and hyperactive are portrayed as having inherent difficulties with no reference to their emotions or efforts to communicate. The child is viewed as suffering from a lifelong disorder that might not be cured but controlled by a diagnosis and subsequent medication. Parents are advised to control their child's behavior and to strive for early diagnosis in order to receive treatment provided by experts. Those who are presented as experts rely on a biomedical model, and in the document, detailed descriptions of medication to correct the undesired behaviors are provided. The value of judgment in the assessment of different symptoms and behaviors that signifies “ADHD” is absent, rather taken-for-granted beliefs were identified throughout the document. A heterogeneous set of behaviors is solely described as a disorder and hereafter it is stressed that the same behaviors are caused by the disorder. In this manner, cause and effects of “ADHD” are intertwined through circular argumentation.

In the Western, industrialized world, there is an ongoing process of individualization in which behaviors, life course, and experiences of human beings increasingly are understood at the level of the individual, whereas the influence of institutions, economy, class, and social crises are underestimated (Beck & Beck-Gernsheim, 2002; Comstock, 2011; Timimi, 2009). In this process, human suffering is seen as inherent in the individual, decoupled from contextual factors and social circumstances (Beck & Beck-Gernsheim, 2002; Gillies, 2005; Timimi, 2011). Parallel to individualization, there is an increasing medicalization in which medicine and the medical profession is expanding its jurisdiction (Comstock, 2011; Kirschner, 2013). When individualization and medicalization become intertwined, there is a tendency to view human suffering, and life crises, as psychiatric disorders that should be subject to diagnostic procedures, rather than as understandable reactions to overwhelming situations (Leo, 2004).

Psychiatric diagnoses are in their nature descriptive and based on subjective judgments (Frances, 2013; Timimi, 2014). Moreover, psychiatric diagnoses are products of classification processes that in turn are influenced by economical and/or political interests, media, researchers, and professional organizations, and as such subjects of change (Frances & Widiger, 2012; Kirschner, 2013; Leo & Lacasse, 2015; Vrecko, 2010). The changing nature of psychiatric diagnoses becomes obvious when it is viewed from a historical perspective. Diagnoses such as hysteria, masturbation, and homosexuality were once defined and treated as disorders, and supported by clinicians and researchers who provided “scientific” support for the diagnoses, and in many cases also dubious interventions (Johannisson, 2015; Mildenberger, 2007). The changing nature of psychiatric diagnoses is also illustrated through the publication of the latest edition of the Diagnostic and Statistical Manual for Mental Disorders (DSM-5) (American Psychiatric Association, 2013). A number of diagnoses in DSM-IV (American Psychiatric Association, 1995) were removed, new ones were introduced, and some had their criteria changed. This is not problematic *per se*. Perspectives and views of knowledge change and in some cases develop for the better. However, if researchers, clinicians, and decision-makers treat psychiatric diagnoses as discrete medical entities, this has implications for how human suffering is viewed and treated (Batstra, Nieweg, & Hadders-Algra, 2014; Brinkmann, 2014). Despite the increasing use of diagnostic procedures and diagnostic categories, the progress in treating the perceived “illnesses” is limited (Middleton, 2015). Middleton further argues that the distinct entities of the diagnosed conditions are yet to be described. Moreover, psychiatric diagnoses function, according to Brinkmann (2014), as a filter through which individuals understand their difficulties, emotions, and themselves; a process that might lead to unnecessary pharmaceutical use. Judging emotional reactions and relational difficulties as a sign of symptoms associated with the diagnosis are likely to hamper other explanations to these shortcomings.

Attention deficit hyperactivity disorder (ADHD) is a paramount example of how human suffering increasingly is viewed as a psychiatric disorder. Freedman (2016) in a discourse analysis of textbooks used in teacher education shows how medical terminology is adopted in school settings, how contextual factors tend to be ignored, and how teachers are encouraged to view themselves as extensions of the medical profession. What seems to have been forgotten is that behaviors perceived as deviating may be reflections of normal variations in personality. Expectations that society places on children of today also contribute to less indulgence toward children who do not fit in. Instead, deviance is medicalized and even if the individual child may not suffer the problem becomes primarily a problem for other people and for the social system. To be observed is that the rate of children being diagnosed with “ADHD” is increasing (Lefever Watson, Arcona, Antonuccio, & Healy, 2014; Moncrieff & Timimi, 2011; Polanczyk, Willcutt, Salum, Kieling, & Rohde, 2014). Pajo and Stuart (2012) in an analysis of self-help books for parents, whose children are perceived as having “ADHD,”^1^ show how these books tend to influence parents to approach their children from a disability perspective. The parents are advised to remind their children what they need to do and control their children's behaviors. “ADHD” is described to co-occur with, for example, difficulties in establishing and maintaining relations with friends, with temper outbursts, and with oppositional behaviors (Lewis-Morton, Dallos, McClelland, & Clempson, 2014; Richards, 2012).

Co-occurring difficulties tend to be depicted as caused by “ADHD,” even though there might be underlying difficulties that lead to symptoms of “ADHD” as well as to other difficulties (Batstra et al., 2014; Richards, 2012). In addition, social and academic problems are a prerequisite for a diagnosis of ADHD. Hence, they are not the results of “ADHD” but part of the definition, that is, impairment criterion. The concept of “ADHD” thus shifts focus from interpersonal dilemmas, problems in the educational system, child-rearing practices, as well as social injustice, and instead focus on dysfunctions in the individual child; a process that serves the interests of the pharmaceutical industry since medication is presented as a solution to the child's difficulties (Antonuccio & Danton, 2003; Mitchell & Read, 2011). The process of medicalization however goes beyond labeling difficulties as disorders. “ADHD,” as well as other psychiatric diagnoses such as depression and substance use disorders, are increasingly classified and/or described as neurological disorders or conditions, thus implying that the experienced difficulties are caused by a known neurobiological dysfunction (Leo, 2004; Leo & Lacasse, 2008; Vrecko, 2010). Classifications cannot, however, be equated with evidence that the classified entities are neurobiological physical disorders/dysfunctions. Moreover, it has been acknowledged that inattentiveness and hyperactivity should be understood with respect to the cognitive and senso-motoric development of the unique child (American Psychiatric Association, 2013; Batstra et al., 2014). Referring to social norms, these behaviors are especially problematic in a society highly valuing academic performance. This has not hindered the depiction of “ADHD” both in the research community and in popular media as a biomedical disorder that must be handled through medication (Bourdaa et al., 2015; Leo & Lacasse, 2015).

Winter, Moncrieff, and Speed (2015) performed a discourse analysis of how “ADHD” was depicted on YouTube by women who had received the diagnosis. Their analysis showed how the women positioned themselves in the biomedical discourse and even promoted the “ADHD” diagnosis. They thus described themselves and their difficulties with a vocabulary adapted from “professionals” and “experts” on “ADHD.” Researchers and scholars from various disciplines have been able to show that it is insufficient to view “ADHD” as a distinct disorder. “ADHD” is, for example, difficult to discriminate from other conditions such as post-traumatic stress disorder (Daud & Rydelius, 2009). Moreover, the symptoms and diagnosis of “ADHD” is laden with contextual values concerning what constitutes a severe symptom and how to draw the line between “normal” and deviant behaviors among children (Hawthorne, 2010). Nevertheless, surprisingly little criticism has been directed toward the biomedical explanation of “ADHD” in popular media and clinical practice.

The biomedical model does not account for any contextual factors such as the influence of social disadvantage, educational systems, traumatic experiences, or the parent–child relationship. DSM and other diagnostic systems do not consider how behaviors may have developed and whether or not they serve a function for the child. Theories other than biomedical theories are thus relevant when attempting to understand hyperactivity and inattention. Several studies have found that “ADHD” is more prevalent in children from families facing serious challenges, such as divorce, low socioeconomic status, mental illness, and/or alcohol or drug abuse (e.g., Chronis et al., 2003; Crittenden & Kulbotten, 2007; Dallos, Denman, Stedmon, & Smart, 2012; Johnston & Mash, 2001; Nigg & Hinshaw, 1998; Pheula, Rohde, & Schmitz, 2011; Rydell, 2010).

One theory that has been proposed in the case of “ADHD” is the attachment theory (Bowlby, 1958) in which the emotional bond between child and caregiver is examined. Children are dependent on the caregiver and must adjust to the care available, meaning that early attachment-related experiences have a profound impact on a child's emotional, social, and cognitive development. Experiences of inconsistent, unresponsive, or insensitive care can imply anxiety, hypervigilance, and distrust in a child (Clarke, Ungerer, Chahoud, Johnson, & Steifel, 2002; Crittenden & Kulbotten, 2007). The child internalizes this capacity to identify and regulate emotions and can subsequently adopt self-regulating skills. Attachment experiences thus play a crucial role for the emergence of emotional self-regulation that in turn has consequences for behavioral regulation (Stiefel, 1997; Waters et al., 2010). In children who are anxious, hyperactivity and inattention can also be a reflection of scanning the environment, which in turn has a self-protective purpose (Crittenden & Kulbotten, 2007). The more energy the child needs to use in order to focus on feeling safe, the less the energy and attention that can be turned to other activities such as schoolwork and friendships.

When a child is perceived as hyperactive, impulsive, and/or inattentive, and an assessment process is initiated, parents’^2^ involvement in the assessment is crucial. A considerable part of the assessment relies on information and ratings that the parents contribute to (Langberg et al., 2010; Rafalovich, 2004; Smith & Corkum, 2007). Moreover, the parents’ involvement is important when interventions are decided on. According to Langberg et al. (2010), parents are expected to take part in interventions, regardless of whether the intervention is centered on medication or behavioral change. The predominant and biased focus on medical treatment, for example, might invoke a process in which parents foreclose toward an illness perspective when they perceive inattentiveness and hyperactivity among children (Lewis-Morton et al., 2014).

Although the process of medicalization is powerful, partly due to the influence of the pharmaceutical industry on public opinion (Antonuccio & Danton, 2003; Mitchell & Read, 2011), professionals as well as parents might be reluctant toward medication as well as to viewing their children's difficulties as symptoms of a disorder (Helle-Valle, Binder, & Stige, 2015). Parents also tend to be concerned with the educational context their children are part of, and might stress that the child's difficulties tend to arise in school situations (Rafalovich, 2004). Moreover, Sciberras, Iyer, Efron, and Green (2010) have shown that parents of children who have been diagnosed with “ADHD” prefer to have a dialogue with health professionals about the specific needs of their children, rather than receive information about “the disorder.” Accordingly, Lewis-Morton et al. (2014) reported that parents of children who were referred for “ADHD” assessment might question the biomedical explanation of “ADHD” and emphasize the child's ability to take responsibility and be self-determined. Altogether, parents appear to a certain degree to be influenced by varying discourses and perspectives, and as a consequence also endorse and sometimes question the biomedical explanation of “ADHD.”

## The epicenter of “ADHD”

Lloyd, Stead, and Cohen (2006) have depicted the USA as the epicenter of the “ADHD” diagnosis. Lloyd et al. (2006) as well as Timimi (2012) describe that diagnosis rates as well as medical interventions are fueled by the pharmacological industry. Furthermore, Comstock (2011) and Mitchell and Read (2011) describe how the diagnosis rate first increased in the USA and some years later the same pattern was seen in, for example, the United Kingdom. Researchers and policy makers in the USA seem to influence perceptions of “ADHD” as well as attitudes about treatment throughout the world. Accordingly, an increasing emphasis on “ADHD” as a biomedical disorder that needs medication in, for example, Norway and Denmark, respectively, have been reported by Pedersen (2015) and by Brinkmann (2014). Pedersen (2015) further acknowledges how the developing emphasis on biomedical markers and medication mimics that in the USA.

Since the view of “ADHD” in the USA, according to the research literature, influences how the “disorder” is depicted and treated in other parts of the world, the purpose of this paper was to investigate what information concerning “ADHD” the National Institute on Mental Health (USA) present officially on their website. As parents are influenced by discourses on “ADHD” and are also involved in interventions when a child receives a diagnosis, our priority goal was to examine how “ADHD” and children perceived as having “ADHD” are described, and how the information was used to provide advice to parents. More specifically, the main goal was to identify, by the use of a discourse analytical approach, naturalized and taken-for-granted assumptions. Influenced by Jörgensen and Phillips (2012), we formulated our main critical questions as: Which understanding of the “ADHD” discourse is *taken for granted* and which understandings and *alternative discourses* are not acknowledged?

## Method

### A discursive approach

Discourse analysis is a method that concerns what talk or text is doing^3^ (Edwards, 2005; Edwards & Potter, 1992). One corner stone in this approach is to shed light on how the phenomenon of interest is described and how it is argued that one representation or explanation should be perceived as superior to another (Edwards & Potter, 1992). Potter (2003) and Potter and Edwards (2001) draw on three theoretical principles of discursive psychology. The first principle defines discourse as both constructed and constructive. It is *constructed* as the building up of words, categories, and repertoires, and so on, which portrays a special version of the world. But it is also *constructive* because using the words, categories and such produces the perceived world. The second principle implies that discourse is action-oriented, meaning that we are acting out within a social arena when writing or talking. The third principle acknowledges that a discourse is always situated; words or writings are situated as it takes place in an institutional setting or within a “particular argumentative framework” (Wiggins & Potter, 2008).

The discourse analysis performed here concerns the role argumentation plays in forming actions among those individuals who read the text. We lean on Edwards (2005) who argues that discursive psychology should be understood as an analysis of how agency is downplayed through the use of passive forms. Discursive psychology concerns how language is applied in argumentations of particular points and version of things, even though a variety of potential perspectives are at hand (Edwards, 2005). We would argue in line with Jörgensen and Phillips (2012) that it is only by constantly looking at perspectives or discourses, which are excluded, that the social consequences of a specific, dominating discourse could be visualized. According to the theory of Laclau and Mouffe (1985), a discourse is always temporary and hence its structure can be challenged and transformed. Hegemony in Laclau and Mouffe's theory implies that there is social consensus, by which the real interest of people is masked (Laclau & Mouffe, 1985). Not even a discourse with a hegemonic status, defined as a closure temporarily, is however completely fixed and so competing discourses can violate the hegemony by the articulation of alternative perspectives.

*Data: Online information concerning “ADHD.”* The data material comprised online information and advice about “ADHD” under the headline: “What is Attention Deficit Hyperactivity Disorder (ADHD, ADD)?” published by National Institute of Mental Health (USA). It was gathered from the website of the institute; www.nimh.nih.gov/health/publications/attention-deficit-hyperactivity-disorder/index.shtml and downloaded on October 25, 2015. Three parts of the text were selected for the discourse analysis. (1) The introductory part, as this sets the tone of the whole text and was considered important for the comprehension of the remaining parts. (2) Those parts of the text that were specifically addressed to parents. (3) Etiology and pathology of “ADHD” with reference to a number of different symptoms and behaviors.

*Discourse analytical steps*. As pointed out by Yardley (1997), language and context have a deep influence on meaning. To be able to grasp what it means to be given a diagnosis like “ADHD” we must understand the language and the context in which the labeling occurs. Particular attention was therefore given to how language is used to structure practices concerning inattention and hyperactivity as well as beliefs concerning how parents should approach the child who is perceived as inattentive and hyperactive. Throughout, the following queries directed the analysis: (1) What rhetorical means are used to describe inattention and hyperactivity? (2) How are the children, who are perceived as inattentive and hyperactive described in the text? (3) What rhetorical means are experts using in order to provide advice to parents concerning inattention and hyperactivity?

*Ethical considerations*. When adopting a discourse analytical approach, the researcher's responsibility also implies to be sensitive to those ethical issues that need to be considered in the investigation. Both questions that are raised to the text and the text itself must be evaluated from an ethical frame of reference. Discursive analysis is a technical and analytical activity rather than a way to provide understanding of individuals and their subjective experiences. Since the method, by definition, implies that arguments are decomposed, problematized, and even questioned, the method should be used with caution when studying individuals who, in any way, are disadvantaged or marginalized. There is a risk that we as researchers place ourselves in a superior position by making use of the individuals’ narratives not telling them that their talk is going to be decomposed and scrutinized. An alternative purpose of discursive methods is however to analyse how communities of, for example, researchers and professional organizations use language on a “macro level” (Seymour-Smith, 2015). Such communities are not underprivileged but rather are in a superior position, holding the power to and influence people as well as other organizations (Seymour-Smith, 2015). Seen from an ethical perspective, to analyse and problematize language used by organizations in superior position should be a core issue in society.

## Results and discussion

It is not clearly stated in the first part of the document to whom the information on “ADHD” is provided. Further down, it appears that the information concerns both parents that suspect that their children are suffering from “ADHD” as well as parents whose children already have received a diagnosis. In this way, parents who perceive their child as inattentive and/or hyperactive become directed toward “ADHD” as an explanation for the perceived problematic behaviors of the child. Thereby, the focus is pointed in the direction of “ADHD” as the most likely answer to the parents’ worries and questions.

### Categorization of “ADHD”

Although “ADHD” is characterized as a floating spectrum of symptoms, it is transformed into a distinct entity that appears clearly defined solely by its name. While it is difficult to define the dividing line between normal and abnormal, the label “ADHD” appears sharp and exact and explanatory in itself. The use of the word “severe” makes the reader observe characteristic differences between the normal and the abnormal child. The definition of abnormal behaviors is, however, a result of social decision-making, and a sharp line between normal and abnormal behaviors is illusory as pointed out by Börjesson (1999) and Winter et al. (2015). Nevertheless, attempts to engage the reader in a critical debate about what constitutes “severe” behaviors are lacking in the analysed document. “Inattention, hyperactivity and impulsivity are the key behaviors of ADHD. It is normal for all children to be inattentive, hyperactive or impulsive sometimes, but for children with ADHD, these behaviors are more severe and occur more often.”

An image of “ADHD” as a legitimate medical disorder is established by the first sentence: “ADHD is one of the most common childhood disorders.” The repeated use of the term “disorder” together with a number of references to brain imaging and brain chemicals creates an impression of a chronic and long-term disability. A detailed and clearly defined description of “ADHD” seems nevertheless hard to concretize. The subtle language reveals the incompleteness of the medical discourse: “To be diagnosed with the disorder, a child must have symptoms for 6 or more months and to a degree that is greater than other children of the same age.” Since social and relational aspects of children's behaviors, emotions, and reactions are toned-down, the reader gets the impression that the described difficulties are causal consequences of “ADHD.” Nothing is mentioned about circumstances within the family that might lead to the child being “out of control”: “Parents may first notice that their child loses interest in things sooner than other children, or seems constantly ‘out of control’.” “Often, teachers notice the symptoms first, when a child has trouble following rules, or frequently ‘spaces out’ in the classroom or on the playground.” No remarks in the text, or references, are to be found concerning the organization of the school in order to understand why the child has troubles following rules or “spaces out.” Alternative discourses are thus excluded. It is stated in the document that “ADHD can be mistaken for other problems.” It is further stated that: “For example, adults may think that children with the hyperactive and impulsive symptoms just have disciplinary problems.”

The discourse of causation leaves no room to question the legitimacy of “ADHD” and so the reader is assured that “ADHD” has a biomedical origin. More specifically, explanations to behaviors such as inattentiveness, hyperactivity, and impulsivity are delayed maturation of the child's brain: “Brain imaging studies have revealed that, in youth with ADHD, the brain matures in a normal pattern but is delayed, on average, by about 3 years. The delay is most pronounced in brain regions involved in thinking, paying attention, and planning. More recent studies have found that the outermost layer of the brain, the cortex, shows delayed maturation overall, and a brain structure important for proper communications between the two halves of the brain shows an abnormal growth pattern. These delays and abnormalities may underlie the hallmark symptoms of ADHD and help to explain how the disorder may develop.” Presumably, after this declaration, some parents get the impression that “ADHD” is a diagnosis with objective criteria. A critical reader however would question why a delay in maturity becomes equivalent with a disorder. In addition, average group differences concerning “ADHD” or any other condition say little about the unique individual (Falkum, 2008).

In fact, as shown in the document, there are no biological markers, environmentally defined categories, or objective tests to distinguish “ADHD” as a discrete condition. Rather, diagnostic criteria are subjectively interpreted from the behavior of the child: “No single test can diagnose a child having ADHD. Instead, a licensed health professional needs to gather information about the child, and his or her behavior and environment.” Professionals as well as teachers and parents are involved in the evaluation and examination process, and subsequently in the diagnostic process. Rafalovich (2004) as well as Batstra et al. (2014) report that professionals rely on parent's accounts of their children in the diagnostic process. Parents are however influenced by, for example, popular media and information provided by actors from the pharmaceutical industry. They might compare their child with how children with “ADHD” are characterized and come to the conclusion that the child suffers from “ADHD” (Lewis-Morton et al., 2014; Pajo & Stuart, 2012). Gonon, Konsman, Cohen, and Boraud (2012) found that newspaper articles “put forward scientific findings to defend the view that ADHD is a neurological disease mainly caused by genetic factors and that psychostimulant treatments are safe and effective” (p. 9). The newspapers however failed to report new scientific findings. This means that findings that initially were claimed to be facts later were refuted in scientific journals, but this never reached the lay public. The authors dispute that the reason for the biased information from newspapers is that they most often publish initial findings in research on “ADHD.” As a consequence, not only the lay public but also clinicians and researchers might be influenced by the erroneous media coverage (Gonon et al., 2012).

Although the expected behavior of being “out of control” is absent, the child might nevertheless meet the criteria for a diagnosis, as exemplified here: “They may sit quietly, but they are not paying attention to what they are doing. Therefore, the child may be overlooked, and parents and teachers may not notice that he or she has ADHD.” The criteria of inattention thus seem hegemonic. Since inattention is a considerably varying concept, according to the document it concerns both being easily bored and switching from one activity to the other and moving slowly, “ADHD” is a subject that varies considerably. Both the child with hyperactive behaviors and the child who is “quiet and well-behaved” are candidates for an “ADHD” diagnosis. There is thus a circular argument in that ADHD is defined according to the presence of particular behaviors which the diagnosis is then proposed to explain. Furthermore, the behaviors associated with ADHD seem hard to define: “Children mature at different rates and have different personalities, temperaments, and energy levels. Most children get distracted, act impulsively, and struggle to concentrate at one time or another. Sometimes, these normal factors may be mistaken for ADHD. ADHD symptoms usually appear early in life, often between the ages of 3 and 6, and because symptoms vary from person to person, the disorder can be hard to diagnose.”

### The problem child

The idea that children suffer from inherent medical disorders diverts attention away from problems that are contextual, such as, for example, problems within the school or within families. Bad feelings in parents are exemplified as stemming from the child's problems and not from family dynamics or other contextual reasons. Parents are comforted by the assumption that they need education about “ADHD” and help to handle negative feelings: “Before a child is diagnosed, frustration, blame, and anger may have built up within a family. Parents and children may need special help to overcome bad feelings. Mental health professionals can educate parents about ADHD and how it impacts a family.” The reader is taught that if the child receives special help, bad feelings caused by “ADHD” can be overcome. In the NIHM document, bad feelings in parents are depicted as a result of the child's inherent inattention and hyperactivity. It is reasonable to assume that parents feel bad if their children have difficulties. However, such feelings might as well be reactions that arise between individuals who are closely related to each other according to Clarke et al. (2002) and Crittenden and Kulbotten (2007).

No observable strengths and positive characteristics in the child are mentioned in the document. When an alternative explanation is raised, it still concerns the inner state of the child indicating signs of psychiatric disorder: “Has anxiety or depression, or other psychiatric problems that might cause ADHD-like symptoms.” Although researchers (e.g., Saul, 2014; Timimi, 2011; Timimi & Leo, 2009; Visser & Jehan, 2009) have put forward a serious critique of the dominant medical discourse, doubts about the validity of “ADHD” are ignored in the NIMH document. What are the reasons to exclude potential contextual or relational factors that could contribute to the child′s so-called severe, excessive, and inappropriate behavior, or his/her depression or anxiety? Contextual factors are portrayed simply as potential enhancers of inherent “ADHD” symptoms—“the social environment might contribute to ADHD”—a remark that serves to underline that “ADHD” is an inherent entity. The tendency to underestimate contextual factors as well as difficulties in relationships and instead view human suffering as inherent in the individual has been raised and problematized (e.g., Beck & Beck-Gernsheim, 2002; Bronfenbrenner, 1986; Comstock, 2011; Timimi, 2009). However, in the absence of any discussion of contextual factors, the reader is left with the impression that “ADHD” is the direct cause of children's inherent difficulties. The individual is portrayed as being detached from contextual factors such as the family, the neighborhood, and the community. Attempts to examine what the child has been through or what the child strives to communicate are absent. There is no regard for whether the child has been affected by a significant and sudden change, such as the death of a family member, divorce of parents, or parent's job loss. There is no regard for the child's home environment. On the contrary, the focus is on finding a psychiatric label that describes the child.

### Experts and recommended treatments

In the document, those who advocate the biomedical discourse are portrayed as experts who have the “correct” knowledge about “ADHD” and hold the power to choose a proper language whenever this label is introduced to the lay public or elsewhere. In their role as experts, they should explain how children with “ADHD” could be best understood and helped. A reference, attached to the document, gives the impression that there are strong reasons to believe that parts of the brain are involved in the pathogenesis of the disorder. “A study of children with ADHD found that those who carry a particular version of a certain gene have thinner brain tissue in the areas of the brain associated with attention. This research showed that the difference was not permanent, however, and as children with this gene grew up, the brain developed to a normal level of thickness. Their ADHD symptoms also improved.”

The biomedical model is portrayed as the evident paradigm, since about a third of the document concerns medication. Professionals who do not adhere to the biomedical model thus implicitly become positioned as unable to help children with “ADHD.” “With treatment, most people with ADHD can be successful in school and lead productive lives. Researchers are developing more effective treatments and interventions, and using new tools such as brain imaging, to better understand ADHD and to find more effective ways to treat and prevent it.” Alternative discourses that acknowledge other understandings and solutions to the children's difficulties are thus excluded. It is assumed that expert professionals are willing to assist children who meet the criteria for an “ADHD” diagnosis. Children perceived to have ADHD need to be evaluated in order to determine whether they qualify for special education services. “Once your child has been evaluated, he or she has several options, depending on the specific needs. If special education services are needed and your child is eligible under the Individuals with Disabilities Education Act, the school district must develop an ‘individualized education program’ specifically for your child within 30 days.” An early identification is stressed to be crucial. “Recognizing ADHD symptoms and seeking help early will lead to better outcomes for both affected children and their families.” The outcome and the future for afflicted children who do not receive help at an early stage are, as this sentence illustrates, not so promising. “These symptoms can make it difficult for a child with ADHD to succeed in school, get along with other children or adults, or finish tasks at home.”

The biomedical discourse assumes that there is a consensus among professionals on how to interpret the behaviors of the child, which means that pharmacological treatment is the preferred intervention. Graham et al. (2011) describe that various drugs that are prescribed to children with “ADHD” might be connected to, for example, cardiovascular risks, tics, and suicide-related events, and the authors underline that the safety of children should be focused. The lack of consensus regarding whether it is safe to offer medical treatment to children diagnosed with ADHD is however not mentioned by NIMH. Instead, medication is described as a safe treatment: “Under medical supervision, stimulant medications are considered safe.” Possible side effects of medication that pose serious threats to children's health are, nevertheless, mentioned in the document: “The most commonly reported side effects are decreased appetite, sleep problems, anxiety, and irritability.” “A few children develop sudden, repetitive movements or sounds called tics.” “The medications may lead to possible cardiovascular (heart and blood) or psychiatric problems.” Serious, although rare, side effects are also described: “a review of data suggested that ADHD patients with existing heart conditions had a slightly higher risk of strokes, heart attacks, and/or sudden death when taking the medications.” A follow-up study of long-term physical effects after pharmacological treatment authored by Vitiello et al. (2012) is included in the reference list: “A recent follow-up found that, over a 10-year period, children with ADHD who were treated with methylphenidate had, on average, higher heart rates compared with children who received other treatments. That this effect on heart rate could be detected even after years of use suggests that the body does not get completely used to stimulants. Children taking stimulants over the long term should be monitored regularly for potential cardiovascular complications.”

Non-medical treatments, such as behavioral training and educational interventions, are discussed briefly after a lengthy description of brands of medication that are available for the afflicted child; “Behavioral therapy aims to help a child change his or her behavior. It might involve practical assistance, such as help organizing tasks or completing schoolwork, or working through emotionally difficult events.” Three pages of the document are devoted to describing medical treatments, a half page is devoted to describing psychotherapy, and one and a half page is devoted to giving advice to parents.

### Advice to parents

Information and advice given to parents are typically presented as aiming at controlling and changing the child's behaviors: “If your teen breaks rules, your response should be as calm and matter-of-fact as possible. Punishment should be used only rarely. Teens with ADHD often have trouble controlling their impulsivity and tempers can flare. Sometimes, a short time-out can be calming.” The categorization of children assumed to suffer from “ADHD” leads to generalizations. Through the diagnosis, the child is distinguished from other children and may incorporate a new identity, shaped by the characteristics of “ADHD.” Hence, the diagnosis functions as a filter through which individuals understand their difficulties, emotions, and themselves, a process that has been described by Brinkmann (2014). It is assumed that when the teenager concerned is not behaving in accordance with culturally sanctioned rules, parents should focus on handling the “ADHD” symptoms. Alternative interpretations of problematic behaviors in children are absent in the text. For example, potential choices to consciously break rules are not elucidated, neither is the agency of the child taken into consideration. Parents are not encouraged to perceive their children as unique individuals who are communicating and expressing their emotions. Parental skills training are described as: “Parenting skills training helps parents learn how to use a system of rewards and consequences to change a child′s behavior. Parents are taught to give immediate and positive feedback for behaviors they want to encourage, and ignore or redirect behaviors they want to discourage.”

The diagnosis is portrayed as a relief for the children as well as for adults who receive a diagnosis later in life since this ought to provide them with an explanation for their difficulties and in turn allow them to handle their problems more effectively. As it is put forward that children should be diagnosed as early as possible, a good parent is a parent who understands that they should help their child to receive a diagnosis in order to prevent future problems. “For some adults, a diagnosis of ADHD can bring a sense of relief. Adults who have had the disorder since childhood, but who have not been diagnosed, may have developed negative feelings about themselves over the years. Receiving a diagnosis allows them to understand the reasons for their problems, and treatment will allow them to deal with their problems more effectively.” Personal advantages for the parents are that they will have an explanation for the child's difficulties.

### Conflicting discourses

Although behavioral training and educational interventions are mentioned in the NIMH document, the inventors are categorizing “ADHD” as a biomedical disorder with medication as the first-choice treatment. Phenomena such as dysfunctional relationships between parents as well as stress and illness in the family can lead to inconsistent and/or insensitive parenting which in turn can result in hypervigilance, fast responses, and inability to regulate emotions (Crittenden & Kulbotten, 2007). For a family who is facing serious challenges, it might be difficult for the parents to be sensitive to the child's signals and needs, and caring responses can thus be inconsistent or dysfunctional (Storebø, Darling Rasmussen, & Simonsen, 2016). Furthermore, behaviors associated with “ADHD” can serve a self-protective purpose for the child (Kreppner et al., 2001).

In the document, there are no attempts to scrutinize any aspect of the “ADHD” diagnosis. Questions that could be raised are: Who and what made the children feel like failures in the first place? How can acceptance of a dysfunction be associated with a more positive development? Seen from the viewpoint of a psychosocial discourse, a diagnosis can be perceived as transporting blame to the child presuming that the child is living in a vacuum. Diagnoses currently are depicted as beneficial since they counteract stigma connected to mental distress. At the same time, according to Timimi (2009), medicalization and diagnostic classification might increase stigma connected to mental distress when those who suffer are viewed as chronically disabled. Leo and Lacasse (2015) as well as Mitchell and Read (2011) imply that those who are likely to benefit most from the view of mental distress as a chronic condition are pharmaceutical companies providing medication for lifelong disorders such as “ADHD.” Moreover, as the attention is foremost directed toward the behavior and the condition of the child, there are no needs for the experts to consider family situation, the school, the society, or any other circumstance. Topics of interest in a psychosocial discourse are thus not taken into account, that is, investigating children living under social and cultural pressure requires consideration of contextual factors.

A discourse is repeatedly constructed by the exclusion of other possible interpretations that belong to the field of discursivity, a term originated from Laclau and Mouffe's (1985) discourse theory. In order to fill the gap, we wish to cast light on conflicting discourses perceived to be powerful in their own rights to compete with the now prevailing discourse. Inattention and/or hyperactivity, like any other human phenomenon, needs to be understood with respect to contextual factors rather than as representing a medical entity. There are good reasons to inquire whether a child who is perceived as inattentive and/or hyperactive in preschool should be assessed with respect to “ADHD,” or whether the organization of the preschool should be the object of assessment. For example, Helle-Valle et al. (2015) reveal that professionals working with preschool children report that inattentiveness and hyperactivity might be the children's response to a lack of structures and resources. Contributing and interacting factors involved in children's perceived misbehavior could, for example, be studied from the perspectives of two well-known theories; Bronfenbrenner's ecological model of development and attachment theory. According to Bronfenbrenner (1986), contextual factors involve the family, the neighborhood, and community including, for example, peer groups, school, and social networks, as well as public policy. One reason for the interest in the attachment theory in relation to “ADHD” is that the theory assumes that early experiences with caregivers are important for the emergence of self-regulatory skills (Mikulincer, Shaver, & Pereg, 2003), that is, by soothing the child, the caregiver can help the child regulate and stabilize intense emotions and also support the child to identify and understand emotional experience. According to Singh (2011), it can be questioned if ADHD is a stable, universal disorder, or rather “a convenient catch-all category” (Singh, 2011, p. 895). On the contrary, although to some extent the diagnosis is culturally relative, Singh assumes that the ADHD diagnosis can be seen as valid as long as diagnostic practices pay attention to the environment and culture in a systematic and flexible way. The goal is to reach beyond reductionist reasoning and incorporate a more complex model. Some of these conclusions come from a study where it was found that in children diagnosed with ADHD in the UK, aggressive behaviors were more pronounced than in children in the USA. Singh proposes a model that resembles a social constructionist interpretation of how human behavior is influenced and shaped through life.

### Methodological considerations and validity

In searching for the prevalence of discourses in the field of “ADHD,” it became clear that the biomedical discourse dominated, and under its wings has made room for a conglomerate of different medical and pharmacological research branches. The discursive method enabled us to entail a critical approach to “taken-for-granted knowledge” maintained by this dominant discourse. The discursive method also made it possible to raise crucial questions with reference to the agenda of the NIMH's information to parents who needed guidance for being able to carry on their role as parents. Validity in discourse analysis can be determined by the examination of coherence in what has been presented. To establish validity, it must be evident that different aspects of the analysis are in line with the discourse (Potter & Wetherell, 1987). Fruitfulness is another concept used for establishing discourse validity; that is, the framework of the analysis has explanatory value and contributes with new perspectives. Focus on coherence in the analysis of the NIMH online information for parents was set on exploring the meaning of the text and what it meant implicitly for both parents and those who received an “ADHD” diagnosis. Considering how different research approaches most often contribute with new and important knowledge, the one-sided perspective on children's “aberrant” behavior must be perceived as a sign of weakness by those researchers and clinicians whose philosophy of science and tradition are committed to social constructionism. Having this and fruitfulness in mind, we added to the text two well-known psychological/ecological frameworks that could be seen as candidates for an alternative discourse in the field of “ADHD.”

## Summary and conclusion

There has been an escalation of biomedical understanding of children's behavioral and emotional problems and a subsequent increase of drug prescriptions for such behaviors (e.g., Polanczyk et al., 2014). Seen from a discourse perspective, it is possible that the more powerful and resourceful one particular scientific direction becomes, the less space is left for alternative, scientific pathways. However, the intensified focus on biomedical foundations to mental suffering will not lead to a clearer view on what kind of support the children need in order to mature on their own conditions. What does it mean for the self and the identity of a vulnerable child that all parties involved agree on the reasons behind the child's incomprehensible behavior? Furthermore, to learn at an early age that the solution to your problem is depending on medication might give rise to a lifelong reliance on drugs. As researchers and clinicians, we must ask what the social consequences are for the present taken-for-granted understanding of children's perceived misbehavior.

It is possible that children identify with and even become attached to their diagnosis, as they in their daily interactions are encouraged to inform other people about the diagnosis. Individuals can, for various reasons, take advantage of an illness or a condition. So-called neuropsychiatric diagnoses have the capacity to relieve suffering to shortcomings and in some cases even provide rewards, such as medication or permission for sick leave (Carone, Iverson, & Bush, 2010; Dige, 2010). Singh (2011) reported that almost all children with ADHD who took part in her study in the UK admitted that they had used their diagnosis as an excuse. However, the children also expressed an ambivalence about their exploitation of ADHD. In the short perspective, the advantage linked to the diagnosis can be experienced as favorable, but in a longer perspective it may not be socially beneficial for the individual.

What consequences will an increase of medicalization of human problems infer on how we conceptualize relationships and social life? To diagnose peoples' vulnerability foremost as biomedical disorders may lead to marginalization. Humans are social beings and a great deal of our psychological distress might originate from social and close relationships early in life. Instead of making the window to “normality” shrink we need to broaden our views on human growth and human conditions.

## Notes

1 In this paper, “ADHD” is consistently written with quotation marks to illustrate that the term should be questioned rather than viewed as representing a medical entity.

2 The term parents is applied in this work since it is used in the investigated data with reference to caretakers who not necessarily are the child's parent.

3 In this paper the term text is used, since the analysis concerns a written document.
